# Tie-Bar Elongation Based Filling-To-Packing Switchover Control and Prediction of Injection Molding Quality

**DOI:** 10.3390/polym11071168

**Published:** 2019-07-09

**Authors:** Jian-Yu Chen, Chun-Ying Liu, Ming-Shyan Huang

**Affiliations:** 1 Bachelor’s Program of Precision System Design, Feng Chia University, 100, Wenhwa Rd., Seatwen, Taichung City 40724, Taiwan; 2 Department of Mechatronics Engineering, National Kaohsiung University of Science and Technology, 1 University Rd., Yanchao Dist., Kaohsiung City 824, Taiwan

**Keywords:** clamping force, filling-to-packing switchover, injection molding, process control, tie-bar elongation

## Abstract

Filling-to-packing switchover (also called V/P switchover) is critical for assuring injection molding quality. An improper V/P switchover setting may result in various defects of injection-molded parts, such as excessive residual stress, flash, short shot, and warpage, etc. To enhance a consistent molding quality, recent V/P switchover approaches adopt cavity pressure profiles requiring sensors embedded in mold cavities, which is invasive to mold cavities and more expensive. Instead of using cavity pressure sensors, by working with the most popular screw position switchover control, this study hereby proposes a novel approach of tuning V/P switchover timing using a tie-bar elongation profile. In this investigation, a dumbbell testing specimen mold is applied to verify the feasibility of the method proposed. The results show that the mold filling and packing stages can be observed along the tie-bar elongation profile, detected by mounting strain gauges on the tie bars. Also, the characteristics of the cavity pressure are similar to those of the tie-bar elongation profile under a proper clamping force condition. Moreover, the varying process parameter settings which include injection speed, V/P switchover point, and holding pressure, can be reflected in these profiles. By extracting their characteristics, the application of the V/P switchover is proved to be realistic. This research conducted an experiment to verify the proposed V/P switchover decision method based on the tie-bar elongation profile. The result showed that the fluctuation of the part’s weight corresponding to a slight change of the barrel’s temperature from 210 °C to 215 °C can be successfully controlled with this method. Besides, the maximum clamping force increment extracted from the tie-bar elongation profile was found to be a good indicator for online monitoring of the reground material variation.

## 1. Introduction

**Figure 1:**
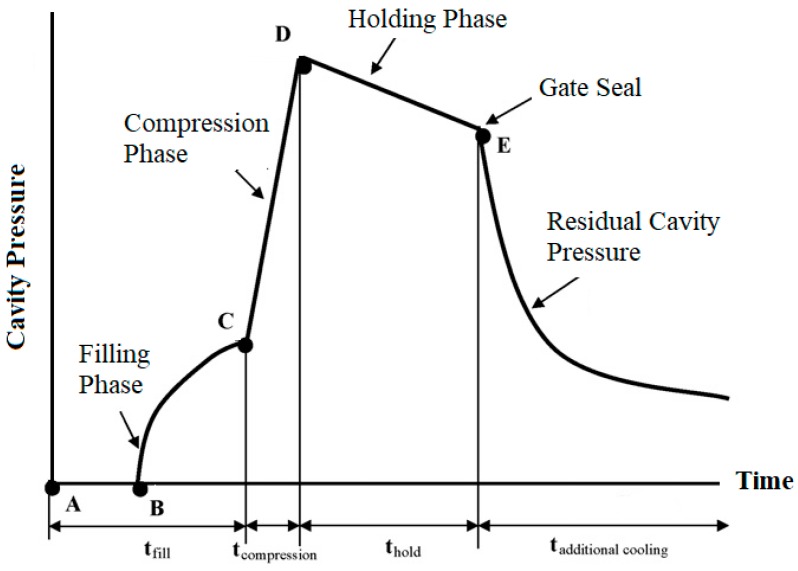
Typical cavity pressure profile.

Injection molding has been broadly used to fabricate components in many industries, such as in automobiles, electronics, sports goods, medical devices, and optical lenses. Injection molding is a cyclic process that comprises of four stages: (1) mold closing and clamping; (2) filling, compressing, and holding; (3) cooling and concurrently plasticizing for the next cycle; and (4) mold opening and ejecting. In stages 2 and 3, the filling, compressing, holding and cooling of molten polymer within cavities have been paid much attention in past studies, since they determine the quality of the injection molding parts [1,2,3]. Particularly, this includes main process parameters such as injection speed and pressure, holding pressure and time, melt and mold temperatures dominate the injection molding qualities, e.g., part appearance, geometrical dimensions, mechanical properties, etc. The behaviors of molten polymer within cavities can be observed by investigating the cavity pressure profiles [4]. Figure 1 shows a typical cavity pressure profile, the pattern from Point A to C represents the filling stage, in which molten polymer enters the mold cavities, following this the cavity pressure is gradually increased based on the injection pressure applied. The filling phase is completed at Point C, where the cavity is only volumetrically filled by the molten polymer without being compressed. The compression process then begins, and the pressure increases rapidly to its peak value at Point D, which has the greatest impact on the resisting mold clamping force introduced by the clamping mechanism in the whole injection molding process. The molten polymer within the cavity is then maintained at a set pressure during the holding phase. Additional molten polymer can be packed into the cavity to compensate for the plastic shrinkage caused by cooling, thus ensuring that the mold is completely filled. This process continues until the gate is frozen, as shown at Point E. This is followed by the final cooling phase, and it continues until the end of the cycle. During this phase, the melt solidifies gradually as the coolant that circulates within the cooling channels in the mold removes heat. The cooling and solidification rates determine the rate while the cavity pressure decreases.

Due to increasing demand for injection molding quality, a proper setting of main processing parameters such as injection speed and pressure, melt and mold temperature, holding pressure and time as well as the cooling rate dominate the results. Particularly, the switching time from mold filling to molding holding, also called filling-to-packing switchover or V/P switchover, is crucial in determining part quality. Notably, the control strategy of an injection molding machine to proceed to mold filling and mold holding is distinct. For mold filling, molten polymer is compressed to fill cavities with a constant flow rate beneath a limited injection pressure to protect machine and mold. In contrast, in mold holding, molten polymer fills mold cavities with a constant holding pressure while the holding speed is limited. This holding stage aims to compensate for the volume shrinkage of the polymer due to cooling.

**Figure 2:**
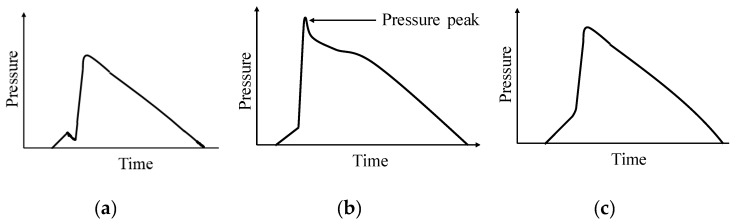
Two typical cavity pressure profiles by filling-to-packing (V/P) switchover: (**a**) early; (**b**) later; (**c**) proper.

A proper V/P switchover time setting is essential in achieving good consistency of the injection-molded parts. If the V/P switchover time is too early, the injection pressure, as shown in Figure 2a may be insufficient to compress the molten polymer into the mold’s cavities, resulting in a short shot, sink mark and warpage, etc. On the contrary, a later V/P switchover time may lead to overfilling and generate excessive cavity pressure as shown in Figure 2b. Improper switchover timing may create serious residual stress/flash on injection-molded part, or even a severe mold deformation and excessive tie-bar elongation. Therefore, a proper V/P switchover time setting not only assures that the molten polymer performs a complete mold filling, but also generates sufficient compression of the molten polymer in the cavity. The cavity pressure profile under a proper V/P switchover is smooth, as shown in Figure 2c.

The cavity pressure profile and its repeatability clearly influence the quality of the molded part, especially its mass, dimensional stability, mechanical behavior, and surface quality. Many studies have proposed that the cavity pressure profile can be used to maintain product quality and help control the machine during the injection-molding process. Besides, other studies indicated that a method to maintain a high yield rate from molding is to reproduce the cavity pressure curve in every shot. Based on these studies, the ideal process parameters have been selected in the past study [4], so the corresponding ideal cavity pressure profile is explored and reproduced by the machine in subsequent shots. Seeing that inconsistent V/P switchover settings can significantly affect the cavity pressure profile, they must be controlled adequately.

**Figure 3:**
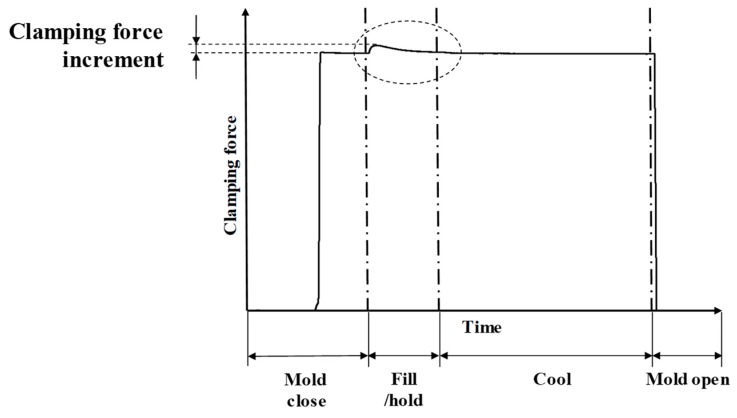
Typical clamping force profile during one cycle of injection molding.

Even though the V/P switchover control based on cavity pressure signals is a well-recognized method, it is costly and inconvenient concerning the installation of pressure sensors in injection mold. In comparison, a strain sensor mounted on the tie bar can present the dynamics of the clamping force during injection molding process. Figure 3 shows a typical clamping force profile in the injection molding process, in which the cavity pressure in the packing stage increases sharply, resulting in mold separation and immediately leading to additional tie-bar elongation, generating a further clamping force increment on the clamping force profile. Choosing the tie-bar elongation profile for V/P switchover control has two major advantages: (1) the profile can be easily detected by a strain sensor mounted on the tie bar, which is free of being invasive to the injection mold. Once the sensor is installed, it is applicable to various injection molds attached to the same clamping mechanism; (2) tie-bar elongation enables the monitoring of the clamping condition and helps in identifying a proper clamping force value to set [5]. In injection molding, the magnitude of the clamping force may affect the quality of the plastic parts. For instance, a small clamping force may produce defects such as flashes and poor geometrical accuracy, whereas a large force could result in insufficient air venting during mold filling/packing, leading to the generation of short shot. Traditionally, clamping force is set at the highest machine specification, which may lead to additional energy consumption. Moreover, heavy loading at the tie bars is detrimental to the durability of processed molds and the machine itself. Therefore, the clamping force is suggested to be set at a proper value for assuring injection molding quality and preventing potential damage on the machine and mold [6]. In this study, we aim to investigate the feasibility of quality monitoring based on the characteristics of tie-bar elongation, further develops a decision rule for V/P switchover in injection molding.

## 2. Literature Review

The rheological property of molten polymer in the injection molding process is dependent on the shear, temperature and pressure and is essentially manifested in the viscosity of the molten polymer. The viscosity determines the flow behavior of the resin through the mold, and therefore has a significant effect on the quality of the molded components. There are three major factors that determine the viscosity of molten polymer, namely (1) the raw material, e.g., the type of material, the rheological characteristics of the material, the humidity conditions, and batch-by-batch variations; (2) the plasticizing effect, which is affected by the geometrical design of the injection screw, the screw rotational speed, the back-pressure, the feeding rate, the barrel temperature and the metering time; and (3) the injection parameter settings, e.g., the injection speed, the mold temperature, the melt temperature, the pressure, and the cooling time. Conventionally, the behavior of molten polymer flow to cavities is hard to visualize, so the process of injection molding acts as a black box to operators. However, with advanced sensing technology, much physical information about molten polymer within the cavities can be revealed [7,8]. For example, the cavity pressure profile detected by a cavity pressure sensor can reflect the variations of molten polymer quality during the mold filling process [9,10,11,12,13]. Regarding V/P switchover control, Kazmer et al. [14] compared the effects of the V/P switchover setting point on molded part quality with seven different methods, including: (1) screw position; (2) injection time; (3) machine pressure; (4) nozzle pressure; (5) sprue pressure; (6) cavity pressure; and (7) cavity temperature. They concluded that methods based on screw position and machine pressure are both sufficient in most injection molding cases, providing quality consistency for the injection-molded parts. Moreover, the application of cavity pressure sensors and temperature transducers are also feasible to develop a V/P switchover decision for the injection molding quality control. Huang [4] revealed a novel V/P switchover control method based on the cavity pressure profile by integrating a simple grey model, GM (1,1), to immediately predict the volumetric-filling point in each shot. The experiment’s results proved the validity of seeking a proper V/P switchover. To obtain a precise switchover control, many factors concerning sensor locations and expense are involved.

**Figure 4:**
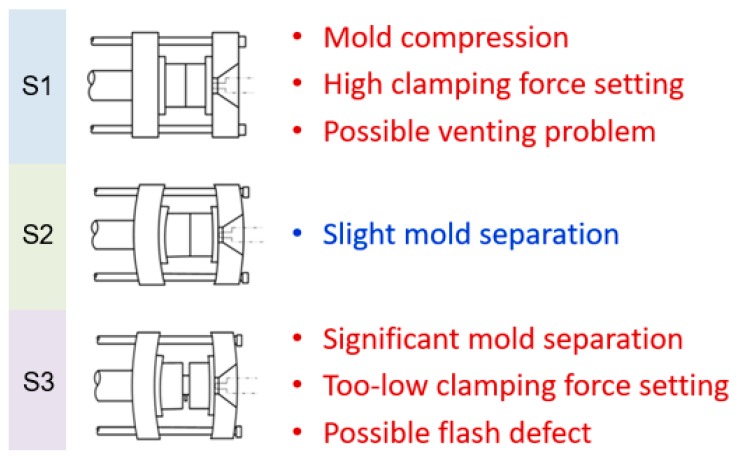
The extent of mold separation under various clamping force set values.

As molten polymer fills the cavity, the cavity’s pressure increases. When the melt nearly fills up the cavity and finally transfers to the compression phase, cavity pressure increases sharply. This phenomenon results in mold separation and immediately leads to tie-bar elongation [5]. Accordingly, there is a clamping force increment on the measured clamping force profile [5], a sufficient clamping force is required to prevent mold separation resulting from excessive cavity pressure as melt fills the cavity. Basically, the extent of mold separation can be classified into three statuses (S1–S3) under various clamping force set values (Figure 4). High clamping force setting results in mold compression (S1) and may then lead to possible venting problems and further potential mold deterioration, tie bar breakage, as well as wear in the clamping component. In contrast, a low clamping force setting creates significant mold separation (S3) during mold filling and packing and then generates possible flash defects. Hence, a proper clamping force which generates slight mold separation (S2) in injection molding is necessary.

**Figure 5:**
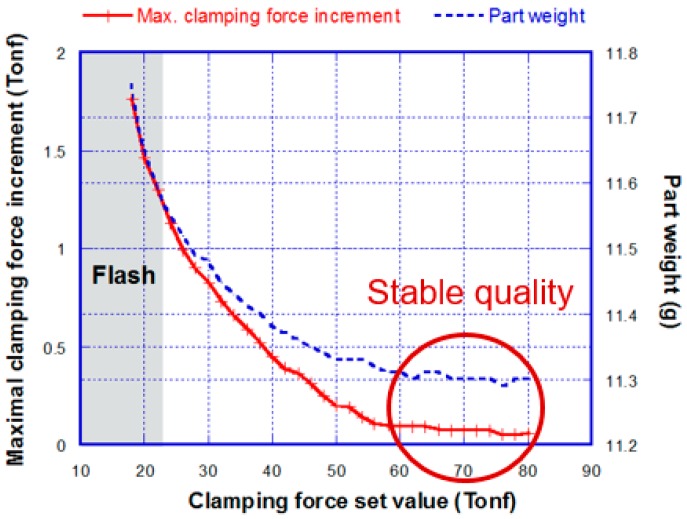
Clamping force set value versus part weight [5].

**Figure 6:**
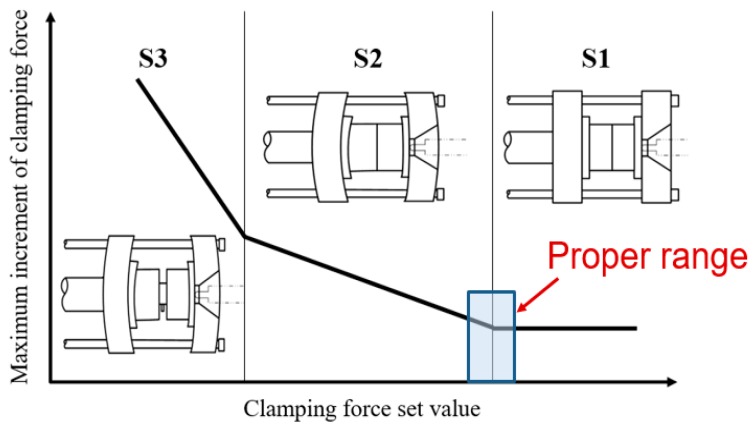
A proper clamping force set value [6].

Huang et al. [5] investigated the effects of mold separation on molded part quality through various melt filling patterns, and found that the molded part quality is crucially affected by whether or not the clamping force is appropriately set (Figure 5). They also founded a method to seek an appropriate clamping force setting based on tie-bar elongation, and verified this with application in a real molding process (Figure 6) [6]. Chen et al. [15,16] investigated the effect of mold separation on molded part quality through a precise linear displacement transducer installed outside the mold plates, and found that the maximum mold separation occurring in the V/P switchover is highly correlated with part weight. Chen et al. [17] conducted an experimental verification of several quality indexes, including peak pressure, viscosity extracted pressure profiles from the system, the nozzle, and the cavity, as well as the clamping force increment extracted from the tie-bar elongation profile. They found that these quality indexes, especially the clamping force increment index, have a strong correlation with the part quality. They also suggested that the clamping force increment index can be used to predict part quality, as it is sensitive enough to indicate the slightly varied V/P switchover and the melt quality fluctuation.

Conventional V/P switchover control in an injection molding machine is mainly based on screw position. Theoretically, the part quality is consistent from shot to shot when the motion control of injection molding machine is accurate and repeatable. However, the melt quality is often disturbed and interfered by extrinsic factors, so the consistency of the molded part from shot to shot proceeding on a precise injection molding machine is still insufficient. In other words, a precisely determined V/P switchover control is essential in compensating for defects of the molded part in order to realize high quality consistency. In recent applications, V/P switchover control based on cavity pressure signals is a well-recognized method, but is highly costed and inconvenient in terms of the installation of pressure sensors in the injection mold. In contrast, a tie-bar elongation sensor is simple to mount on the surface of the tie-bar without invading the mold cavity. Currently, sensors on tie bars are used to measure the surface strain with a press-on sensor directly at the mounting location [16] or ultrasonic sensors [18]. The tie-bar strain sensors, which are similar to bonded strain gauges, can be used to measure the clamping force. The strain gauges are compressed under a stainless-steel protective foil wrapped tightly on the cylindrical surface of the tie bar to be measured. Based on an accurate measurement of the clamping force acting on mold halves during injection molding with the tie-bar strain sensors, an injection molding machine can detect the variation of tie-bar elongation online. Estimation of the minimal clamping force for achieving high injection quality with low energy consumption and machine and mold damage is feasible. This study investigates the feasibility of quality monitoring based on the characteristics of tie-bar elongation, further developing a decision rule for V/P switchovers in injection molding.

## 3. Experimental Setup

**Figure 7:**
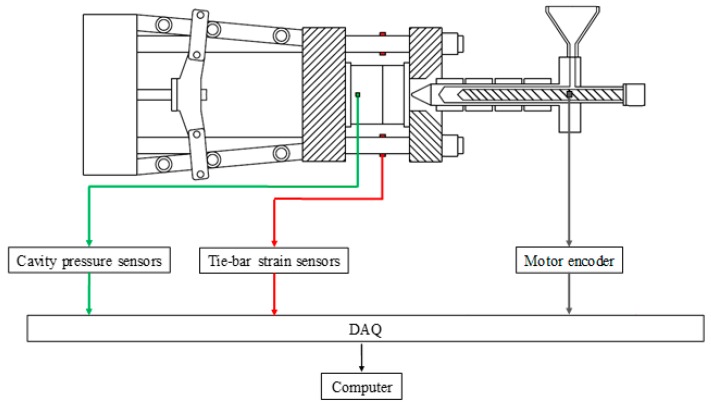
Diagram of the measuring equipment.

**Figure 8:**
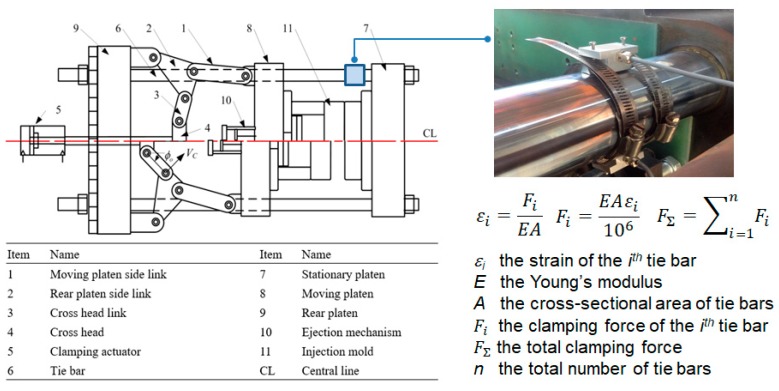
Measurement of clamping force.

The device applied in this research contains an all-electric driven injection molding machine with 100 tonnage clamping force and a 28-mm screw diameter, made by Fanuc Company, Japan. Tie-bar elongation sensors and cavity pressure sensors are separately installed on the injection molding machine and mold (Figure 7), while the strain gauge sensor used to detect the tie-bar elongation is shown in Figure 8. The specification of the sensors applied in this research is listed in Table 1. The tie-bar strain sensors (GE1029) applied to measure clamping force are manufactured by GEFRAN Corp., Germany. The accuracy and repeatability are smaller than ±0.5% full scale and 0.1% full scale, respectively. The strain gauges were pressed under a stainless protective foil wrapped tightly on the cylindrical surface of the tie bar to be measured.

(1) $$\varepsilon \;=\;\frac{F}{EA}$$

(2) $$F=\frac{EA\varepsilon \times 10^{10}}{9.81}\times 4$$

where ε represents the strain of a tie bar in micrometers, *E* is the Young’s modulus of the tie bar and its value is 210,000 kgf/cm^2^, *A* is the cross-sectional area of a single tie bar in mm^2^, and *F* is the clamping force in kNs.

**Figure 9:**
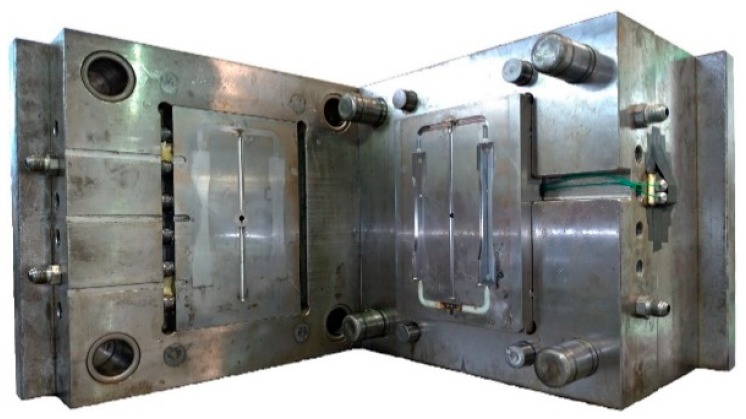
Injection tool with two cavities.

**Figure 10:**
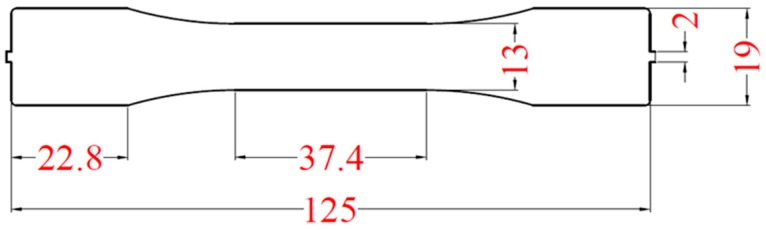
Geometry of dumbbell-shaped specimen.

Figure 9 shows that the configuration of an injection mold has two cavities, allowing molding for a dumbbell-shaped specimen (Figure 10). The geometry of the 1.2 mm thick specimen follows the regulations of ASTM D638. The raw material used in this research is acrylonitrile butadiene styrene (ABS) made by CHI-MEI Corporation, Taiwan (PA756 with melt flow index (MFI) 4.4 g/10 min). The recommended processing operation for melt temperature and mold temperature settings are 180–230 °C and 40–80 °C respectively. In this research, through different level settings of injection speed, V/P switchover and holding pressure, the effect of process parameters on clamping force increment and cavity pressure are observed. In addition, compared to the characteristic of cavity pressure profile, the determination of V/P switchover based on the characteristic of clamping force increment profile was developed. The experimental parameter settings in this study are listed in Table 2, whereas a proper clamping force is set to be 600 kN referring to [6].

## 4. Results and Discussion

### 4.1. Cavity Pressure Profiles Affected by V/P Switchover Timing

**Figure 11:**
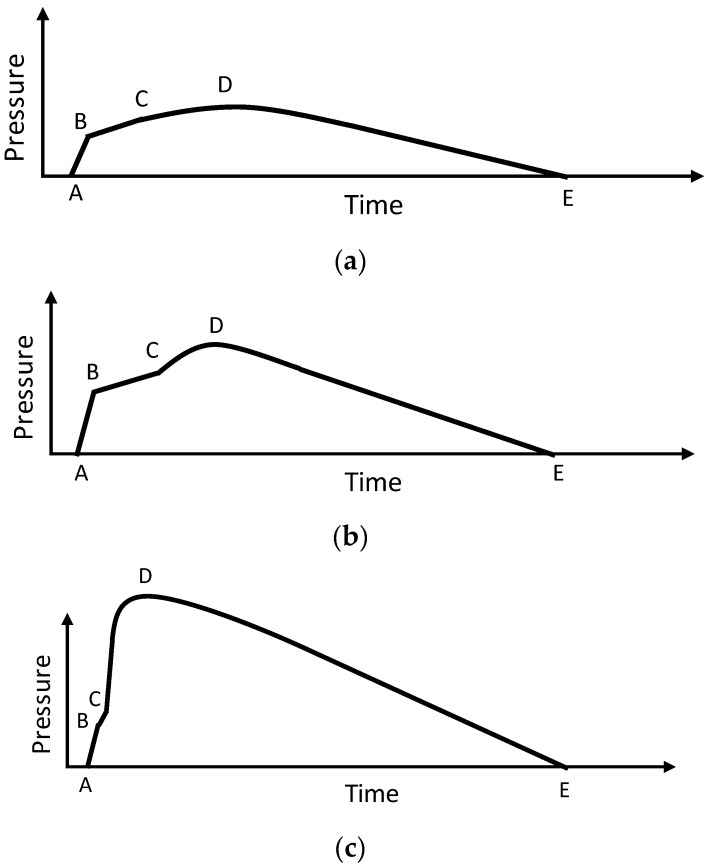
Patterns of cavity pressure profile affected by the V/P switchover timing. Pattern (**a**), Pattern (**b**), Pattern (**c**).

To better describe the cavity pressure profiles affected by V/P switchover timing in the following experimental results, we summarize that there are generally three types of pressure profiles which represent the flowing behaviors of polymer melt during mold filling, packing, and holding process (Figure 11). In Segment A-B, the polymer melt fills the cavity before arriving at the V/P switchover point, and the pressure rises, representing the polymer melt filling the mold cavity with a constant flow rate at the filling stage. After passing through point B, the so-called ideal V/P switchover point, the polymer melt continues to fill in the cavity with constant pressure and limited injection speed until arriving at point C. From point B to C, the pressure profile rises slowly, whereas segment B-C represents the polymer melt filling the cavity from the beginning of the holding stage. Notably, point C represents the polymer melt volumetrically filling in the mold cavity followed by acting compression. After the polymer melt passes point C, the cavity pressure profiles can be classified into three patterns:

(1) Pattern A: Polymer melt performs insufficient or just complete mold filling, in which the compression phenomenon does not clearly exist at the holding stage (Figure 11a). Accordingly, there is no obvious piecewise point appearing after point C in the pressure profile.
(2) Pattern B: Polymer melt sufficiently fills up the whole cavity at the holding stage and performs compression after point C (Figure 11b). Consequently, an obvious piecewise point exists at point C that reflects the compression behavior of the polymer melt.
(3) Pattern C: Polymer melt excessively fills up the cavity after point C in the holding stage, leading to a sudden rise in segment C–D along the pressure profile (Figure 11c). Hence, an obvious piecewise appears at point C, also reflecting an excessive compression behavior among the polymer melt.

Therefore, the slope at segment C-D varies with respect to the level of compression among the polymer melt. Meanwhile, the mold separation reflected is also distinct and consistent with tie-bar elongation. Thus, segment C-D in this research is defined as the polymer melt compression phase. After point D, the cavity pressure curve drops smoothly to point E in the cooling stage, in which the polymer melt is cooled down and shrinks volumetrically in the cavity. Segment D-E is therefore defined as the volumetric shrinkage phase of the polymer melt. As the holding stage is completed, the cavity pressure at point E reflects the over-packing characteristic if excessive compression of the polymer melt occurs.

### 4.2. Effect of Injection Speed, Switchover Point and Holding Pressure on Cavity Pressure and Clamping Force Increment

**Figure 12:**
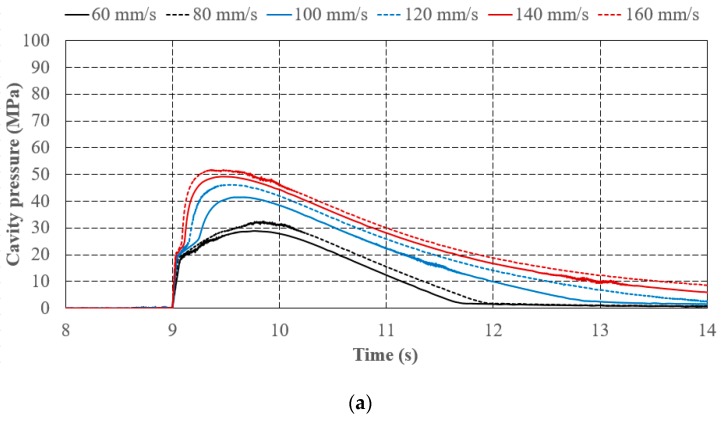

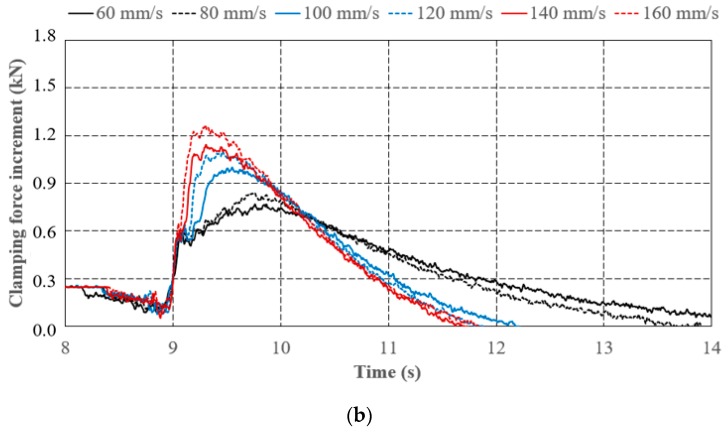
Effect of injection speed on (**a**) cavity pressure and (**b**) clamping force increment.

**Figure 13:**
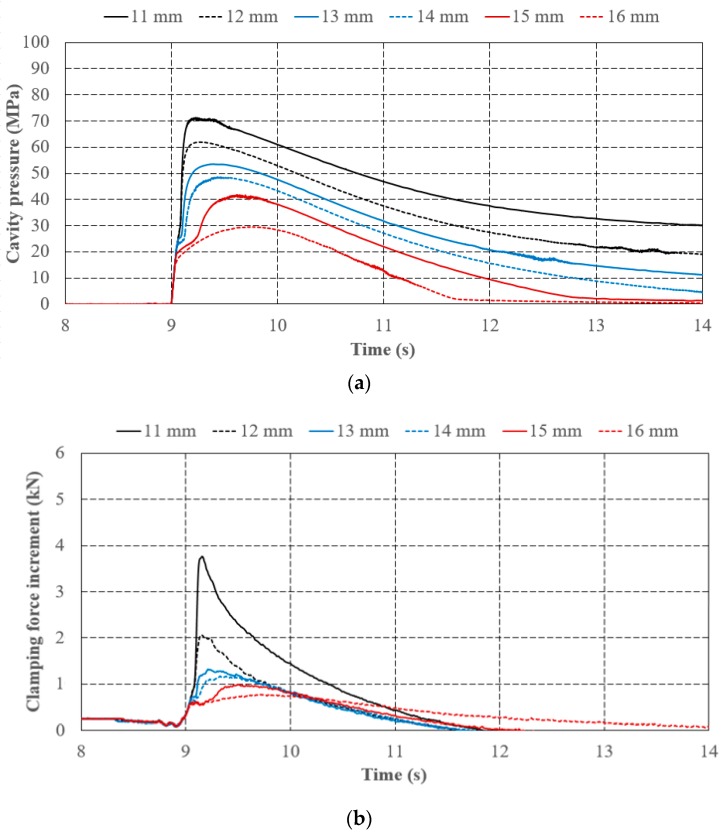
Effect of V/P switchover on (**a**) cavity pressure and (**b**) clamping force increment.

**Figure 14:**
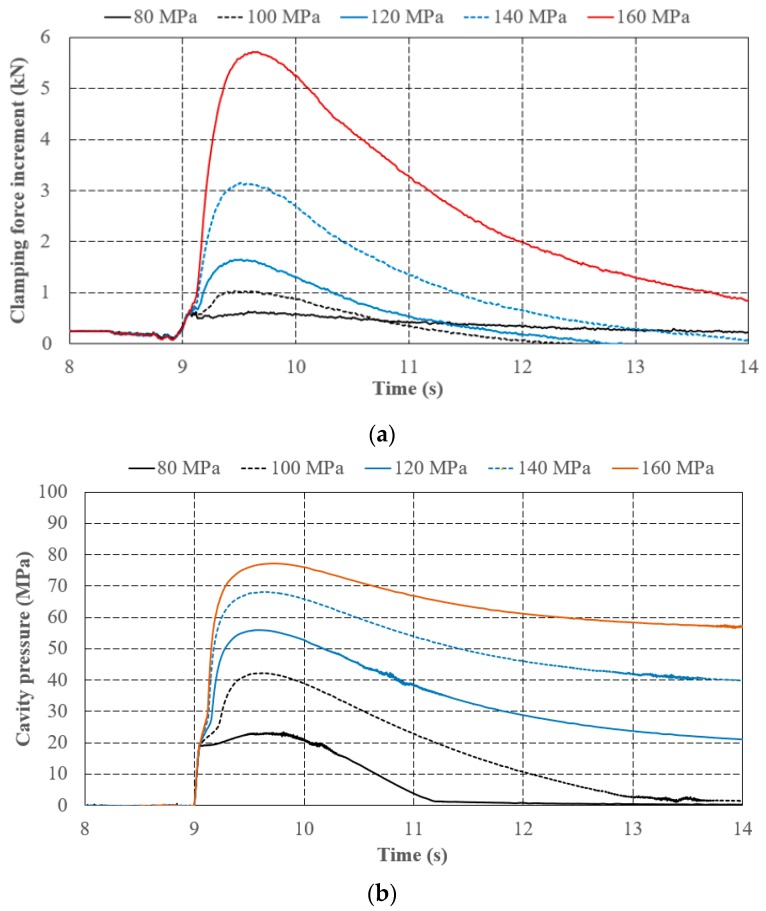
Effects of holding pressure on (**a**) cavity pressure and (**b**) clamping force increment.

Figure 12a,b show the experimental results of varied injection speeds under identical V/P switchover (referring to screw position at 15 mm) and holding pressure (100 MPa) settings. Notably, these sensed pressure and clamping force increment signals at the starting time have shifted to the same starting time for subsequent comparisons (Figure 12, Figure 13 and Figure 14). Comparing the patterns of measuring profiles obtained by the cavity pressure sensor and the tie-bar strain sensor, the patterns of segments A-B, B-C, C-D are most concerned with the V/P switchover in this study (referring to Section 4.1), and the peak value at point D is visually similar to each other. Figure 12a,b show that a higher pressure that forces the polymer melt to fill in the mold cavity appears when the injection speed is increased at the filling phase. Consequently, the slope at segment A-B becomes sharper with increasing injection speed. If the mold cavity is not volumetrically filled at the holding phase, the slope at segment B-C with respect to varied injection speed changes insignificantly. When the polymer melt compression phase is executed at a low injection speed (60 and 80 mm/s in this case), the slope at segment C-D showing the compression behavior is insignificant, which is classified as Pattern A in Figure 11a. The compression phenomenon is relatively obvious with a higher injection speed (100 and 120 mm/s in this case), which is identified as Pattern B in Figure 11b. Also, the piecewise point C appears earlier because more polymer melt is injected into the mold cavity which is due to a large inertia of injection screw motion in high speed. Besides, the peak pressure appeared at point D is also higher. As the injection speed significantly increases to 140 and 160 mm/s, the sudden compression behavior may lead to overshot and the pattern is similar to Pattern C in Figure 11c. In these cases, the patterns of clamping force increment which successfully reflect the mold separation are similar to those of cavity pressure. Table 3 shows quantitative evidence of the characteristic similarities between cavity pressure and clamping force increments with respect to varied injection speeds. Except for the negative slope of segment B-C in the clamping force increment which is obscured in this study, the other slopes of segments, the peak values of the cavity pressure, and the clamping force increment are all increased with increasing injection speed.

Figure 13a,b show the experiment results regarding different V/P switchover point that is referring to screw position with the same injection speed (60 mm/s) and holding pressure (100 MPa). By comparing the patterns of the cavity pressure profile and the clamping force increment profile, their trends are identical to the assumptions mentioned in Section 4.1, i.e., Pattern A for a switchover at 16 mm, Pattern B at 13, 14 and 15 mm, and Pattern C at 11 and 12 mm. To further investigate the effect of the V/P switchover setting on the profiles detected by the cavity pressure sensor and tie-bar strain sensor, the slopes of segment A-B from measuring cavity pressure profiles are visually identical, as an equivalent pressure is required to drive molten polymer to maintain a constant injection speed at the filling phase despite of various V/P switchover points. Point B is getting higher as the V/P switchover point is delayed gradually. It is due to the fact that an increase of screw moving produces more polymer melt packing within the mold’s cavity, so cavity pressure increases. Meanwhile, segment B-C with various switchover point settings becomes shorter, reflecting a quicker beginning of the following compression phase. As for segment C-D, its slope and peak pressure at point D increases when the delay of V/P switchover point is increased. It is because the compression phenomenon of the polymer melt after the V/P switchover point is more obvious since the buffer at the end of the cavity in the filling phase is shortened. Table 4 shows quantitative evidence of the characteristic similarities between cavity pressure and clamping force increments with respect to varied V/P switchovers referring to screw position. The slopes of segment B-C of the cavity pressure, the slope of segment C-D, the peak values of the cavity pressure, and the clamping force increment are all increased with delaying V/P switchovers.

Figure 14a,b show the experiment results of varied holding pressures set with constant injection speed (60 mm/s) and V/P switchover point (referring to screw position at 15 mm). The profile characteristic of clamping force increment is similar with that of cavity pressure. In addition, the profile characteristic of clamping force increment is more significant than cavity pressure. From the measuring profile, the slope and length of segment A-B with different holding pressure settings are the same, and point B also overlapped with identical injection speeds and switchover point settings. After point B, the slopes of segment B-C and C-D become sharper as the holding pressure increases. Notably, the holding phase is engaged with controlled pressure and limited speed in injection molding machines. The peak pressure at point D also becomes higher as the holding pressure increases. After point D, the excessive holding pressure setting leads to over-packing and results in a residual pressure at point E (holding pressure is larger than 120 MPa in this case). Table 5 also shows quantitative evidence of the characteristic similarities between cavity pressure and clamping force increment with respect to varied holding pressures. The slopes of segment A-B are slightly changed due to identical V/P switchover settings in these cases. As to the slopes of segments B-C, C-D, the peak values of cavity pressure, and clamping force increment, all increased with increasing holding pressure as expected.

By introducing the three major process parameters, namely injection speed, V/P switchover point, and holding pressure, factors affecting part quality in injection molding, such as changes of the cavity pressure profile and clamping force increment profile is proved to be correlated in this study. Table 6 shows quantitative evidence in which most characteristics are all highly correlated except for the slope of segment B-C being obscure, particularly when the clamping force increment profiles are reflecting with varied injection speeds. Generally, the cavity pressure profile, which is identified as a good indicator of the variation of injection-molded part quality, can also be reflected in the clamping force increment profile. To seek a consistent injection molding quality, the peak value of the clamping force increment can also be used as a quality indicator and controlled by adjusting the process parameters. The following paragraphs will describe an example of using the V/P switchover point to shot-by-shot control the peak value of clamping force increment to maintain a consistent part weight.

### 4.3. Control of Part Weight by Changing V/P Switchover Point

**Figure 15:**
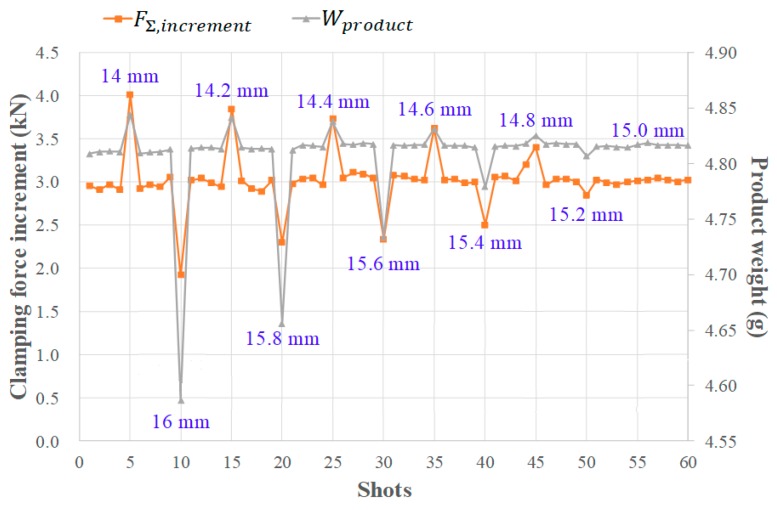
Sensitivity of V/P switchover point to part weight.

Initially, the sensitivity of changing V/P switchover point to part weight is proved by experiment conducted in this study, whereas the V/P switchover point referring to screw position is purposely changed in a sequence of 15 mm, 14 mm, 16 mm, 15 mm, 14.2 mm, 15 mm, 15.8 mm, 15 mm, 14.4 mm, 15 mm, 15.6 mm, 15 mm, 14.6 mm, 15 mm, 15.4 mm, 15 mm, 14.8 mm, 15 mm, 15.2 mm, and 15 mm. Figure 15 shows a slight change in the part weight is reflected by changing the V/P switchover position, in which the maximal clamping force increment and part weight are highly correlated.

**Figure 16:**
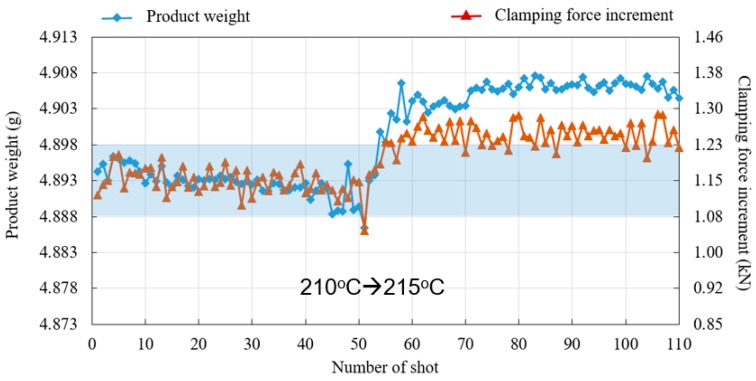
Part weight corresponding to varied barrel temperatures.

**Figure 17:**
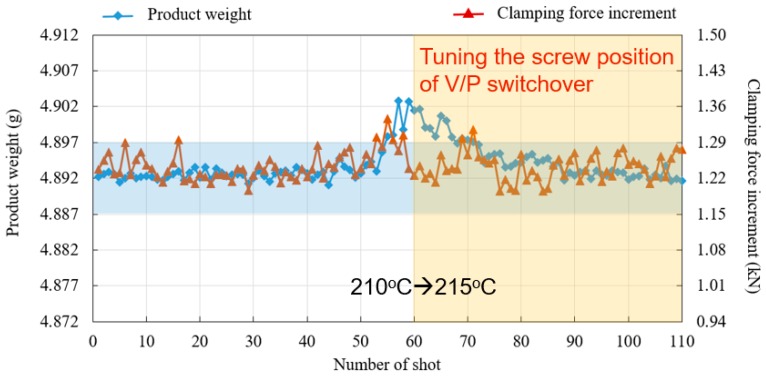
Reduction of part weight variation by V/P switchover point related to clamping force increment.

**Figure 18:**
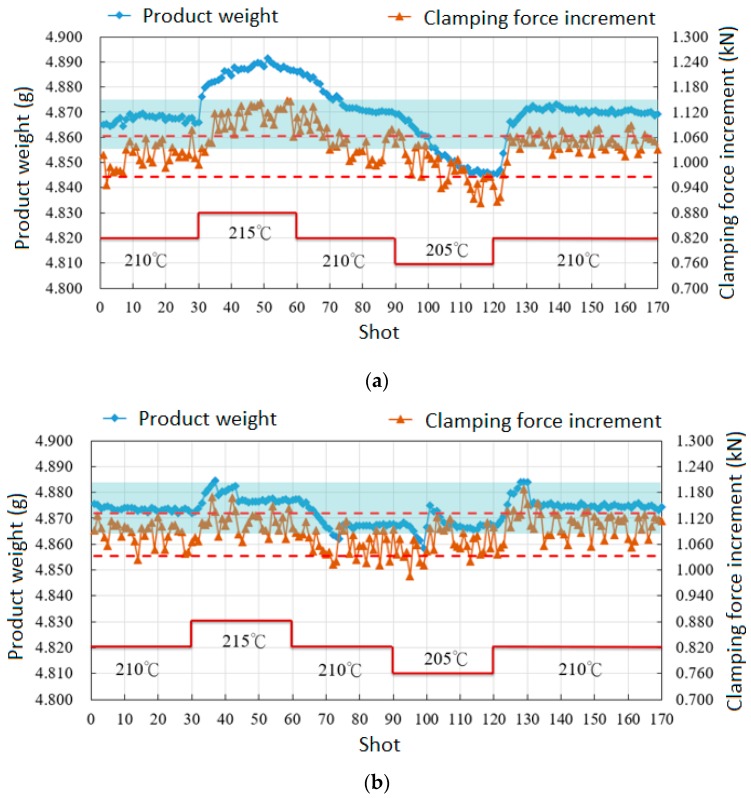
Reduction of part weight variation corresponding a series of barrel temperature change by V/P switchover point referring to clamping force increment: (**a**) original; (**b**) control engaged.

To further prove the feasibility of changing V/P switchover point to part weight, this study conducted an experiment which slightly changed the barrel temperature from 210 °C to 215 °C. The part weight is expected to increase, since a higher barrel temperature enhances the flowing ability of molten polymer when filling the cavity. Figure 16 shows the change of part weight corresponding to varied barrel temperatures, in which both the product weight and clamping force increment increase and run out of the specification (blue block). Figure 17 shows the result of reducing part weight variation with the proposed V/P switchover control method. The screw-position V/P switchover point is further related to the value of the clamping force increment, i.e., when a continuous shot with over clamping force increment is detected, an earlier V/P switchover is performed. In contrast, a later V/P switchover is adjusted. The figure also describes how both the part weight and clamping force increment are tuned back within the specification after a few shots. Furthermore, in a series of barrel temperature variations in a sequence of 210 °C, 215 °C, 210 °C, 205 °C, and 210 °C, Figure 18 shows that the fluctuation of part weight can be successfully reduced with this method, as the original weight range 0.048 g is decreased to 0.027 g.

### 4.4. A Potential Indicator of Clamping Force Increment to Material Fluctuation

**Figure 19:**
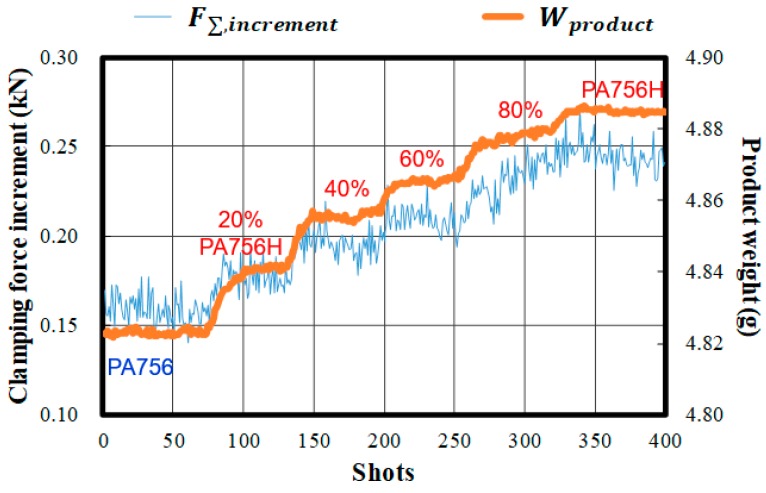
Potential melt quality fluctuations using clamping force increment.

This study further conducted an experiment to verify whether clamping force increment can indicate batch-to-batch variation of the material property. In the experiment, the PA756 pellets (ABS polymer made by Chi-Mei Co.) with an MFI value of 40 mL/10min were gradually replaced with PA756H, which has superior flowing ability with 80 mL/10min MFI. Figure 19 shows the change of the viscosity of the molten polymer induced by a change in the raw material, which can be indicated with the varied clamping force increments. This result proves that the clamping force increment is a possible indicator for online monitoring of the reground material variation.

## 5. Conclusions

Although injection molding technology has been developed for more than one hundred years, developing an intelligent process parameter setting is crucial in satisfying an increasing demand for injection molding quality. An appropriate molding parameter setting dominates part quality. Particularly, it is critical for the V/P switchover point to be free of defects. Hence, this research has developed a novel V/P switchover decision method based on the tie-bar elongation profile. Our experimental studies found that the V/P switchover is crucial in affecting the quality of parts. Defects of over-packing and flashes appeared in injection-molded parts due to late switchover point settings. In contrast, short shot and low density resulted from an early switchover setting. Furthermore, injection speed, V/P switchover point, and holding pressure are most influential to cavity pressure profiles and clamping force increment profiles. In particular, characteristics of clamping force increment profile is found to be similar to that of the cavity pressure profile. Therefore, the decision rule for the V/P switchover point has the potential to be successful based on the tie-bar elongation profile. In addition, the online fluctuation of batch-to-batch processed material can be detected online by monitoring the clamping force increment, calculated from the tie bar elongation profile. It is essential for assuring processing quality and is practical for injection molding.

## Figures and Tables

**Table 1:** Specifications of sensors.

Sensor	Supplier	Type
Cavity pressure sensor	Kistler	6159A
Tie-bar strain sensor	GEFRAN	GE1029
DAQ card	National Instruments	USB-6343

**Table 2:** Process condition settings in this research.

**Fixed Parameters**			
Feeding stroke (mm)	40	Holding time (s)	5
Barrel temperature (°C)	210	Cooling time (s)	15
Screw rotational speed (rpm)	100	Mold temperature (°C)	60
InjectionpPressure (MPa)	180	Clamping force (kN)	600
**Varied Parameters**			
Injection speed (mm/s)	60, 80, 100, 120, 140, 160		
V/P switchover (mm)	11, 12, 13, 14, 15, 16		
Holding pressure (MPa)	80, 100, 120, 140, 160		

**Table 3:** Quantitative characteristics of cavity pressure and clamping force increment with respect to varied injection speeds.

Injection Speed (mm/s)	Cavity Pressure				Clamping Force Increment			
	Slope of seg. A-B (MPa/s)	Slope of seg. B-C (MPa/s)	Slope of Seg. C-D (MPa/s)	Peak Value (MPa)	Slope of Seg. A-B (MPa/s)	Slope of Seg. B-C (MPa/s)	Slope of Seg. C-D (MPa/s)	Peak Value (kN)
60	183.76	14.43 *	14.43 *	28.86	2.46	0.01	0.32	0.78
80	288.67	17.88 *	17.88 *	32.10	2.73	0.61	0.52	0.85
100	348.06	40.79	36.60	41.46	3.07	–0.10	1.18	1.01
120	378.37	65.06	56.90	46.10	4.65	–0.19	1.62	1.12
140	448.68	74.93	67.41	49.18	5.37	–1.14	2.52	1.16
160	485.35	85.72	95.43	51.58	6.07	–1.02	2.84	1.28

* Point C is not significant, and the slopes of segments B-C and C-D are approximated with the slope of segment B-D.

**Table 4:** Quantitative characteristics of cavity pressure and clamping force increment with respect to varied V/P switchover.

V/P Switchover (mm)	Cavity Pressure				Clamping Force Increment			
	Slope of Seg. A-B (MPa/s)	Slope of Seg. B-C (MPa/s)	Slope of Seg. C-D (MPa/s)	Peak Value (MPa)	Slope of Seg. A-B (MPa/s)	Slope of seg. B-C (MPa/s)	Slope of Seg. C-D (MPa/s)	Peak Value (kN)
11	334.94 **	334.94 **	304.46	70.94	4.74 *	4.74 **	32.77	3.83
12	333.24 **	333.24 **	176.30	61.92	4.90 *	4.90 **	13.91	2.09
13	363.63	103.77	92.96	53.48	3.92	0.37	4.45	1.34
14	369.81	59.09	73.06	48.35	3.64	−3.86	2.19	1.18
15	340.56	39.26	42.01	41.44	3.53	−0.50	1.28	1.00
16	331.23	22.01 *	22.01 *	29.48	2.79	−0.04	0.37	0.79

* Point C is not significant, and the slopes of segments B-C and C-D are approximated with the slope of segment B-D. ** Point B is not significant, and the slopes of segments A-B and B-C are approximated with the slope of segment A-C.

**Table 5:** Quantitative characteristics of cavity pressure and clamping force increment with respect to varied holding pressures.

Holding Pressure (MP)	Cavity Pressure				Clamping Force Increment			
	Slope of Seg. A-B (MPa/s)	Slope of Seg. B-C (MPa/s)	Slope of Seg. C-D (MPa/s)	Peak Value (MPa)	Slope of Seg. A-B (MPa/s)	Slope of Seg. B-C (MPa/s)	Slope of Seg. C-D (MPa/s)	Peak Value (kN)
80	362.15	8.00 *	8.00 *	22.97	3.28	−0.86	0.27	0.64
100	361.69	42.06	40.87	42.18	3.15	−0.11	1.51	1.05
120	361.68	87.41	65.93	55.93	3.40	1.15	2.56	1.68
140	361.55	134.35	84.99	68.11	3.53 **	3.53 **	6.09	3.21
160	361.48	143.88	77.45	77.19	3.90 **	3.90 **	9.50	5.83

* Point C is not significant, and the slopes of segments B-C and C-D are approximated with the slope of segment B-D. ** Point B is not significant, and the slopes of segments A-B and B-C are approximated with the slope of segment A-C.

**Table 6:** Correlation analysis between cavity pressure and clamping force increment.

Varied Parameters	Slope of Seg. A-B	Slope of Seg. B-C	Slope of Seg. C-D	Peak Value
Injection speed	0.93	−0.83	0.94	0.99
V/P switchover	0.82	0.58	0.99	0.88
Holding pressure	n/a	1.00	0.76	0.89
